# Adaptive deep learning-based neighborhood search method for point cloud

**DOI:** 10.1038/s41598-022-06200-z

**Published:** 2022-02-08

**Authors:** Qian Xiang, Yuntao He, Donghai Wen

**Affiliations:** 1 School of Electronic and Information Engineering, Beihang University, Beijing, 100191 China; 2 Troops 66133, PLA, Beijing, 100191 China

**Keywords:** Computer science, Electrical and electronic engineering

## Abstract

Point cloud processing is a highly challenging task in 3D vision because it is unstructured and unordered. Recently, deep learning has been proven to be quite successful in point cloud recognition, registration, segmentation, etc. Neighborhood search operation is an important component of point cloud deep learning models, and directly affects the performance of the model. In this paper, we propose a learnable neighborhood search method. This method adaptively chooses an appropriate search method based on the characteristics of each point, thus avoiding the disadvantage of selecting the search method manually. We validate the proposed methods on ModelNet40 dataset and ShapeNetPart dataset, and all the chosen models achieved a performance improvement with a maximum improvement of 1.1%. The proposed method is a plug-and-play technique and can be easily integrated into existing methods.

## Introduction

The rapid development of 3D sensors has increased the demand for point cloud processing technology. At present, point cloud processing technology is widely used in sensor systems such as AR, autonomous driving, and pose estimation^1–3^ In recent years, deep learning methods are widely used in point cloud processing and have achieved good results^4–6^. Because a single point cannot provide enough information to identify a local structure, neighborhood search technology is very important in point cloud analysis. At present, there are two main methods to achieve the objective: one is to search a neighborhood in 3D space, and the other is to search it in feature space.

The difference between the two methods lies in their search space. The former method searches in 3D space, while the latter method searches in feature space. This leads to the formation of different neighborhoods by the two methods. The former method aggregates local information, thus preserving the local topological relationship. In this way, a point cloud is divided into a group of 1-hop subgraphs. In the latter method, proximity in feature space differs from proximity in 3D space, leading to the nonlocal diffusion of information throughout the point cloud. The neighborhood in feature space is equivalent to a group of dynamic n-hop subgraphs from 3D space, which gives the feature space a stronger ability to capture global features. Especially in multilayer systems, affinity in feature space captures semantic characteristics over potentially long distances in the original embedding.

The neighborhood searched in 3D space has clear local topology information, which makes it widely used in point cloud normal estimation, feature extraction, outlier removal and other tasks; examples include PointNet++^7^, PointCleanNet^8^ and D3Feat^9^. Because the neighborhood searched in feature space makes better use of learned features, it has rich semantic information and makes it easier to obtain global information. Therefore, it has a good effect on unsupervised learning, feature retrieval and semantic segmentation, as in GraphTER^10^ DGCNN^11^, and LDGCNN^12^. Many studies have been devoted to improving the flexibility of 3D space searches, such as specifying the search direction^13^ and clustering points to special points^14^ There have also been some studies on building a better feature neighborhood, such as using clustering instead of brute search. Using a data structure to speed up the search is an important improvement direction, such as using KD-Tree^15^or OC-Tree^16^ to speed up the search.

Generally, 3D space searching only depends on local information, so the information it provides is not rich enough, and selecting the neighborhood size is difficult. Feature space searching obtains enough local and global information due to the introduction of long dependency, but the features are not abstract enough in the low-level layers in multilayer networks, which often leads to the neighborhoods in feature space being unstable and having no clear meaning.

For the problem of how to select the search method in multilayer networks, one option is to use 3D space searching to obtain local neighborhoods and aggregate features in the low-level layers and use feature space searching to obtain nonlocal neighborhoods in the high-level layers to enrich the extracted information. This method has been proven to be feasible in experiments^17^, but to the best of our knowledge, there is no clear design principle to find the best search method directly according to different network attributes.

Our approach is to change the selection of the search method into parameters that can be optimized by the model. The method links a neighborhood in 3D space with a neighborhood in feature space. In addition, we assign weights to these two branches that are learned from a point attention feature. We call this “soft-shortcut” ATSearch. Thus, each point can adjust its search tendency adaptively according to its own characteristics.

In summary, the main achievements of this paper are follows:

1. In view of the characteristics of the existing 3D point cloud neighborhood search methods, this paper proposes an adaptive neighborhood search method based on point features and attention module.
2. The proposed method is plug-and-play and can be easily inserted into the existing point cloud deep learning models in many tasks.
3. The performances of proposed method are evaluated on popular 3D shape classification and segmentation data sets. Experimental results show that the proposed method can improve the performance of the models.

The rest of this paper is organized as follows. The “Methods” section introduces the method of the proposed neighborhood search method based on the features of point cloud and different search spaces. The “Results” section verified the effectiveness of the proposed method through experiments. The “Discussion” section studied the proposed search method, robustness-test, visualization and ablation experiments are performed in this section. “Conclusion” are in the last section.

## Methods

### Traditional neighborhood search methods for point cloud

In previous works, the neighborhood searching methods for point cloud can be categorized into two ones, one method is based on the spatial distance between pairs of points, which finds the neighbor points in 3D space, shown in (1). The other method is based on the feature distance between pairs of points, shown in (2).

1 $$\left\{ {\begin{array}{*{20}l} {{\text{d}}_{{{\text{xyz}}}} = \left\| p_{i} - p_{j} \right\| ,\forall p_{j} \in P} \hfill \\ {N\left( {p_{i} } \right) = \{ p^{j} |d_{xyz}^{j} < d_{k} \} } \hfill \\ \end{array} } \right.$$

2 $$\left\{ {\begin{array}{*{20}l} {{\text{d}}_{{\text{f}}} = \left\| f_{i} - f_{j}\right\| ,\forall f_{j} \in F} \hfill \\ {N\left( {{\text{f}}_{{\text{i}}} } \right) = \{ {\text{f}}^{{\text{j}}} |d_{f}^{j} < d_{k} \} ,i = 1, \ldots ,k} \hfill \\ \end{array} } \right.$$

where ${\text{P}} \in {\mathbb{R}}^{N \times 3}$ and $F \in {\mathbb{R}}^{N \times C}$ represent the positions and features of points in the point cloud. $d_{xyz} \in {\mathbb{R}}^{N \times N} , \;{\text{and}} \;d_{f} \in {\mathbb{R}}^{N \times N}$ represent the pairwise distances of points in 3D space and in feature space, respectively. $p_{i} \in {\mathbb{R}}^{3} \;{\text{and}}\; f_{i} \in {\mathbb{R}}^{C}$ represent the center points, and $p_{j} \in {\mathbb{R}}^{3} \;{\text{and}}\; f_{j} \in {\mathbb{R}}^{C}$ are the corresponding neighbor points.

Search methods based on the spatial distance focus on preserving local information, and search methods based on feature distance focus on global information and nonlocal diffusion of information. In point cloud deep learning models, different convolution layers have different requirements for local and nonlocal information^17^. Therefore, manually selecting a search method will limit the performance of the models.

### Proposed adaptive search method for point cloud

**Figure 1:**
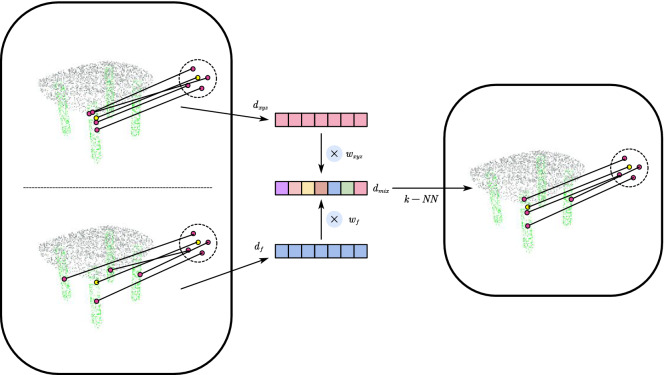
Neighborhood search illustration. Left top: using the distance in 3D space as the search metric. Left bottom: using the distance in feature space as the search metric. Right: The proposed method weights the two metrics by learning from point attention features, then searches the neighborhood using the “mixed” distance. With this method, a point’s neighborhood in the point cloud can be flexibly searched.

Equation (1) and (2) show that the key process of the traditional search method is to calculate the distance between points, In order to obtain the advantages of search methods in 3D or feature space, we propose a new method: attention search (ATSearch), which uses a “soft” shortcut to account for the feature distance and spatial distance when searching neighborhoods. The search method proposed in this paper is a combination of the advantages of the search methods in 3D space and feature space, which is essentially a secondary sampling from the 3D neighborhood based on 3D coordinate distance and the neighborhood in feature space based on the feature distance. So that both local and non-local information can be obtained to further improve the model performances. The differences from traditional search methods are shown in Fig. 1.

3 $$\left\{ {\begin{array}{*{20}l} {d_{mix} = w_{xyz} \cdot d_{xyz} + w_{f} \cdot d_{f} } \hfill \\ {N\left( {p_{i} } \right) = \{ p^{i} |d_{mix}^{i} < d_{k} \} ,i = 1, \ldots ,k} \hfill \\ \end{array} } \right.$$

where $w_{xyz} \in {\mathbb{R}}^{N} \;{\text{and}} \;w_{f} \in {\mathbb{R}}^{N}$ represent the point search preference. It can be easily seen that (1) and (2) are special cases of (3) for $w_{xyz} = 1,w_{f} = 0$ and $w_{xyz} = 0,w_{f} = 1$, respectively. Taking the general case, we can obtain a “mixed” distance $d_{mix}$ by weighting $d_{xyz}$ and $d_{f}$ and then searching the neighborhood on it. The method proposed in this paper can search neighborhoods more flexibly, and it can not only choose how to search neighborhoods according to the characteristics of a point but also enhance the model’s ability to find features across regions. The selection of $w_{xyz} \;{\text{and}}\; w_{f}$ needs to be considered carefully for good performance.

To determine the selection range of $w_{xyz} \;{\text{and}} \;w_{f}$, we first define their basic rules to ensure that the selection is logical: (1) $w_{{{\text{xyz}}}} ,w_{{\text{f}}} \ge 0$. (2) $w_{{{\text{xyz}}}} ,w_{{\text{f}}}$ are negatively correlated.

These are very broad conditions, so we have many choices. One of the simplest choices is to use fixed values as a shortcut, such as $w_{xyz} = 1,{ }w_{f} = 1$. This approach has low computational cost, but it is as inflexible as (1) and (2). On the other hand, we can set $w_{xyz} \;{\text{and}}\; w_{f}$ to be learnable and use a function to normalize the result, but this method has difficulty taking advantage of the characteristics of the point cloud itself. Finally, we choose a generation method to obtain $w_{xyz} ,w_{f}$; specifically, we first obtain the attention feature and then generate the weight from it.

**Figure 2:**
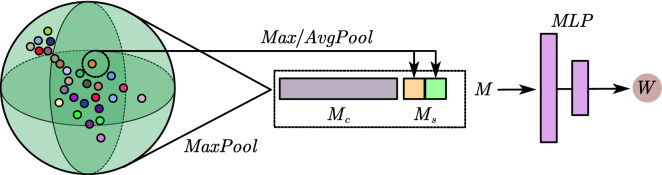
Generation of $w_{d} \;{\text{and}}\; w_{s}$ from point cloud features. $M_{c}$ is the global descriptor of the point cloud, and $M_{s}$ is the point’s local feature. The point attention feature is obtained by concatenating $M_{c}$ and $M_{s}$. Then, an MLP layer is used to generate $w_{d} \;{\text{and}}\, w_{s}$, and softmax is used to normalize the output.

The weight generation step is a two-stage procedure, and is shown in Fig. 2. In the first stage, the attention feature of a point is obtained, and in the second stage, the search weight is obtained from the feature.

4 $$f_{pa} = {{\upvarphi }}\left( f \right)$$

5 $$w_{xyz} ,w_{f} = \phi \left( {f_{pa} } \right)$$

where $f \in {\mathbb{R}}^{C}$ is the point feature and $f_{pa} \in {\mathbb{R}}^{d}$ is the attention feature of the point. $w_{xyz} {\text{and}} w_{f} \in {\mathbb{R}}$ are the search weights.

To obtain the relative features of points, we design a lightweight point attention (PA) operator inspired by Convolutional Block Attention Module(CBAM)^18^. Similar to the CBAM, the PA operator obtains information from channels and space and then generates attention features. The CBAM needs to obtain spatial information from adjacent pixels, but this operation is inefficient in point clouds because point clouds are unstructured. Therefore, the PA operator simply obtains the feature of a single point as the spatial information $M_{s}$ by ${{\upvarphi }}_{1} :{\mathbb{R}}^{N \times C} \to {\mathbb{R}}^{N \times d}$, and the channel information $M_{c}$ is obtained through ${{\upvarphi }}_{2} :{\mathbb{R}}^{N \times C} \to {\mathbb{R}}^{C}$. Finally, $f_{pa}$ is generated by concatenating $M_{c}$ and $M_{s}$.

6 $$M_{s} = {{\upvarphi }}_{1} \left( f \right), M_{c} = {{\upvarphi }}_{2} \left( f \right)$$

7 $${\text{f}}_{{{\text{pa}}}} = {\text{concat}}\left( {M_{s} ;M_{c} } \right)$$

Because ${{\upvarphi }}_{2}$ needs to satisfy the point order invariance, we use $MaxPooling\left( {MP} \right)$ as the selection. In addition, ${{\upvarphi }}_{1}$ chooses MP with ${\text{AvgPooling}}\left( {AP} \right)$.

The pointwise attention feature $f_{pa}$ cannot be used to describe the search weights directly. Therefore, we need to use a function to generate search weights. To define $\phi :{\mathbb{R}}^{d} \to {\mathbb{R}},{\mathbb{R}}$, a simple and logical choice is to generate the initial search weights by learning from $f_{pa}$; then, we use a function to normalize the weights. In this paper, we use a learnable weight matrix $W \in {\mathbb{R}}^{2 \times d}$ to generate the initial search weights and $softmax$ to normalize the search weights:

8 $$\phi \left( {f_{sub} } \right) = {\text{z}} \cdot s{\text{oftmax}}\left( {Wf_{pa} } \right)$$

**Figure 3:**
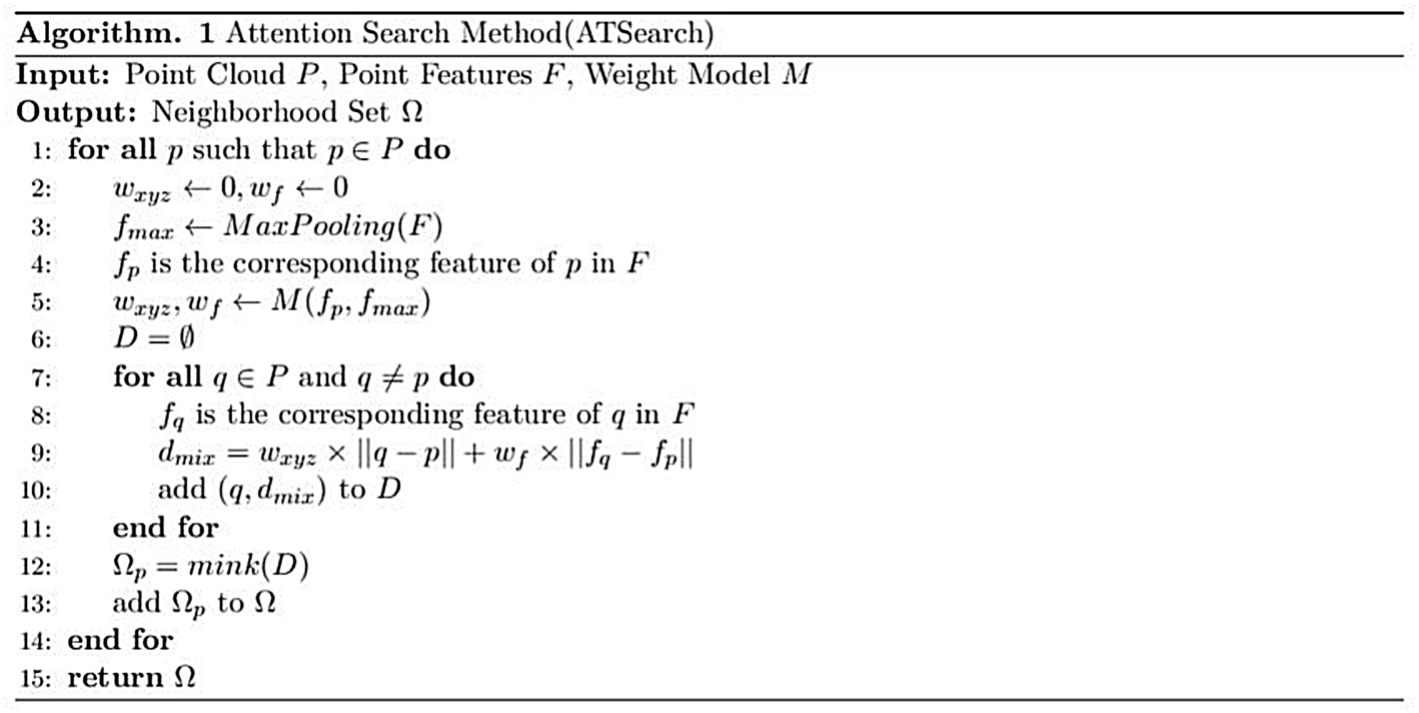
Pseudo code for ATSearch.

And the pseudo-code for ATSearch is shown in Fig. 3.

### Applications in point cloud deep learning models

**Figure 4:**
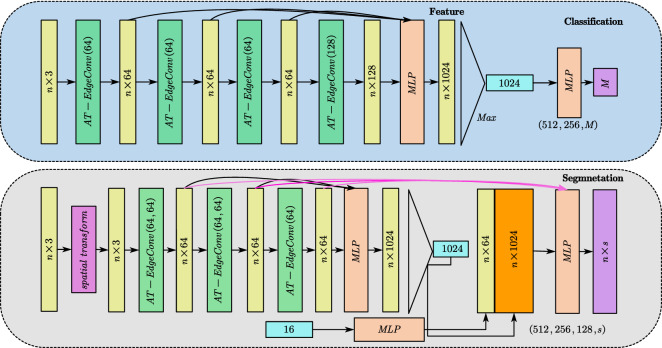
Top: ATDGCNN used for classification task; Bottom: ATDGCNN used for segmentation task. Both of them use the proposed search method in their convolution operator to gather neighbor points.

The proposed search method is plug-and-play, so we just need to change the search operation of models to the proposed ATSearch. We integrate the proposed ATSearch into two basic blocks in point cloud deep learning models: SetAbstraction^7^ and EdgeConv^11^. the overview of DGCNN after using ATSearch is shown in Fig. 4, because the proposed method is plug-and-play, in order to use ATSearch in point cloud deep learning models, we only need to replace the origin neighborhood search operation with ATSearch in each convolution layer.

### Datasets and training details

ModelNet40 dataset^19^ and ShapeNetPart dataset^19^ are used for training and testing in our study. ModelNet40 dataset includes 12,311 CAD models belonging to 40 categories and ShapeNet part benchmark consists of 16,881 CAD models from 17 categories. Points are uniformly sampled from the CAD model surface. During the training period, parameters were updated by the SGD optimizer, with the learning rate set to 0.1. All experiments are implemented using PyTorch 1.5 and models are trained on one Nvidia RTX Titan.

## Results

Tables 1 and 2 show the classification and segmentation performance of different models. PointNet++^7^, DGCNN^11^, LDGCNN^12^, RSCNN^20^ are the models in previous works.

**Table 1:** Shape classification results on Modelbet40 (%).

Model	Accuracy	Accuracy (with ATSeach)
DGCNN	92.4	93.4 (+ 1.0%)
LDGCNN	92.7	93.0 (+ 0.3%)
PointNet++	92.1	93.1 (+ 1.1%)
RSCNN	92.5	92.6 (+ 0.1%)

**Table 2:** Shape segmentation results on ShapeNet Part (%).

Model	Instance mIoU	Instance mIoU (with ATSeach)
DGCNN	85.1	85.5 (+ 0.4%)
LDGCNN	85.1	85.2 (+ 0.1%)

In terms of classification performance, instance accuracy is used as the evaluation metric. The performance of each model is improved when the search method is replaced by ATSearch. Among these test models, the best is PointNet++, which has an accuracy increase of 1.1%, and the accuracy improvement of RSCNN is limited because of it has explicitly encoded the points’ relations. In summary, the classification results prove that ATSearch can improve the accuracy of existing models without changing their structure.

In terms of segmentation performance, we utilized mIOU as the evalution metric, The result shows that our proposed method is also helpful for segmentation tasks, which shows that our search method can extract enough local details and retain global information compared with the search methods that only depend on spatial distance or feature distance.

## Discussion

To further study the proposed search method, robustness-test, visualization and ablation experiments are performed in this section. The baseline model is chosen to be DGCNN, and the dataset is chosen to be ModelNet40. To show the difference in results when using ATSearch, we name EdgeConv as ATEdgeConv and DGCNN as ATDGCNN.

**Figure 5:**
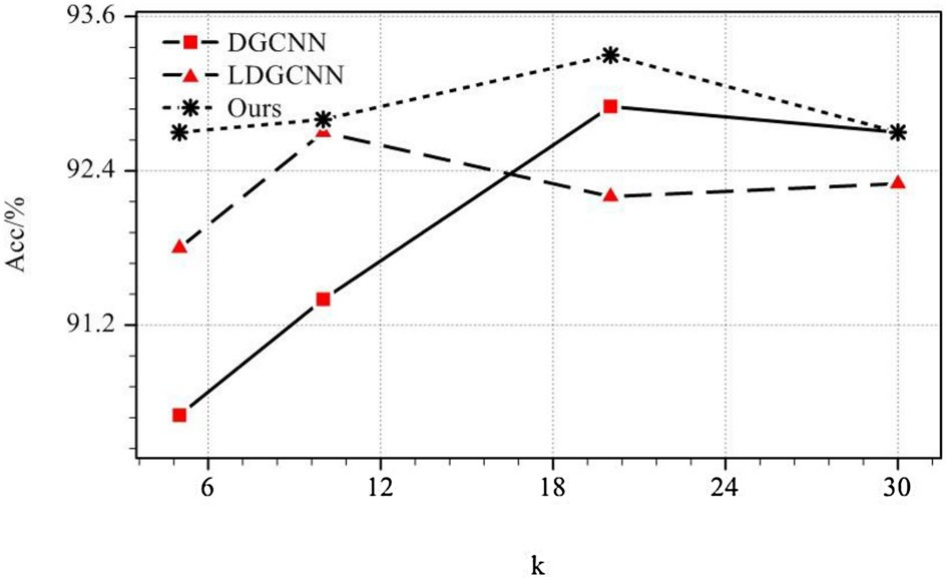
The sensitivity of k used in K-Nearest Neighbor algorithm (k = 5, 10, 20, 30).

The choice of the neighborhood size is the key of the search method, it often directly affects the performance and generalization of the deep learning models. Specifically, in the k nearest neighbor algorithm, the selection of k is an important hyperparameter. The compared results are shown in Fig. 5 and ATDGCNN is significantly more robust than DGCNN and LDGCNN.

To explore the influence of the number of our search operations on the accuracy, ablation study is performed and the results are summarized in Table 3 The addition of ATSearch in any layer can improve the performance of the model, but only when the first two layers are equipped with ATSearch together.

**Table 3:** Ablation study of ATSearch on DGCNN(%), AT ${\varvec{i}}$ stands for using ATsearch at layer ${\varvec{i}}$.

Model	AT1	AT2	AT3	Accuracy
Baseline				92.4
A	√			93.2
B		√		93.0
C			√	93.0
D	√	√		92.5
E		√	√	93.1
F	√	√	√	93.4

There will be a slight performance degradation. This may be caused by the difficulty of matching in the last layer.

**Figure 6:**
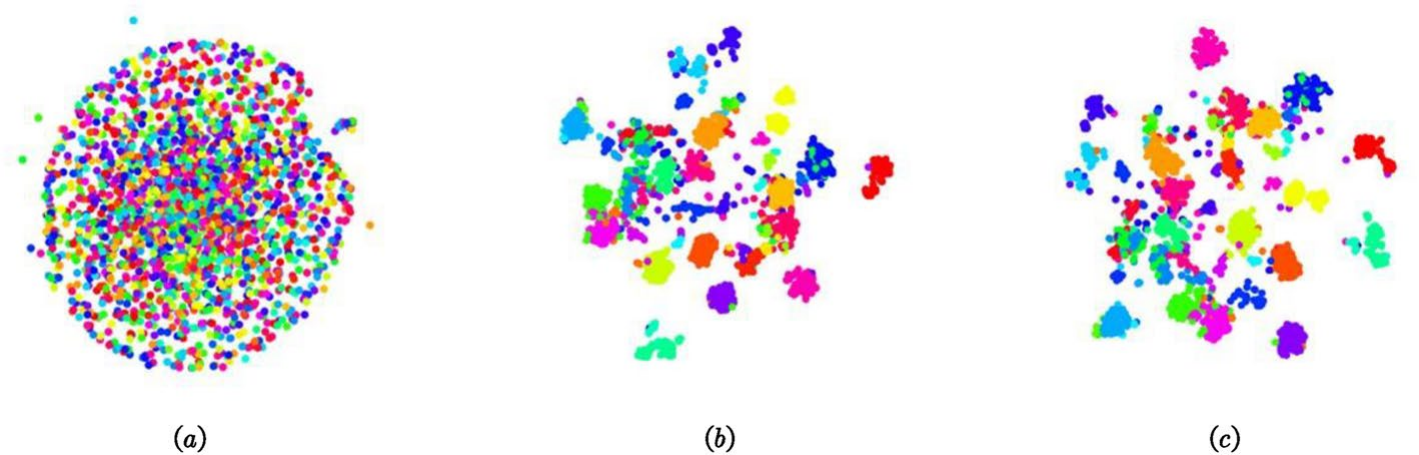
(**a**) Original ModelNet40 dataset. (**b**) DGCNN’s output (c) ATDGCNN’s output.

T-SNE^21^ is utilized to demonstrate the performance of our feature extractor. T-SNE reduces the dimensionality of high- dimensional features to visualize the separability of the features. As shown in Fig. 6, the extracted features are much more discriminative than the original point cloud. Compared with DGCNN, ATDGCNN’s output is even more discriminative.

**Figure 7:**
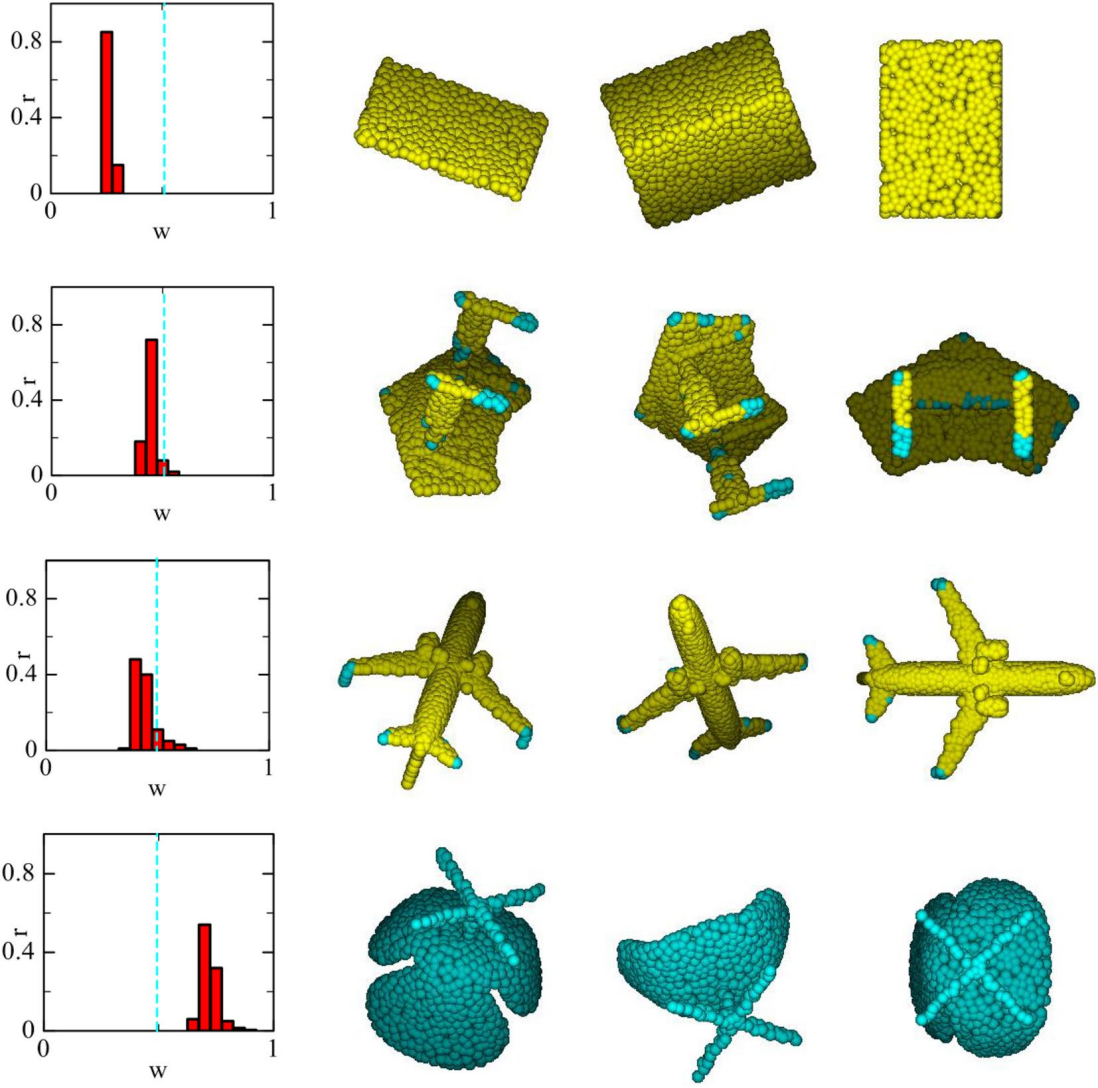
Left: the histogram represents the distribution of search weight in feature space($w_{f}$). Right: In the point cloud, the points in yellow are more likely to search the neighborhood in 3D space ($w_{xyz} > w_{f}$), while the points in cyan are more likely to search in feature space.

Several point clouds are randomly selected from the ModelNet40 dataset and used to visualize the $w_{xyz} \;{\text{and}}\; w_{f}$ distribution of ATDGCNN’s output. It can be seen from Fig. 7 that in different point clouds, the *w* distribution has its own tendency. For example, the more complex the shape is, the more inclined it is to search the neighborhood in feature space. This shows that the characteristics of the point cloud itself have a unique search preference, so it is proven that our method is feasible.

Finally, to explain how the weights $w_{xyz} ,w_{f}$ affect the model performance, we do experiments with fixed weights: (1) $w_{xyz} = 1,w_{f} = 0$, the neighborhood is only sampled from 3D space; (2) $w_{xyz} = 0,w_{f} = 1$. the neighborhood is only sampled in feature space. (3) $w_{xyz} = 0.5,w_{f} = 0.5$. the neighborhood is sampled from both two spaces with equal probability.(4) $w_{xyz} \;and\; w_{f}$, the neighborhood is adaptively sampled according to the point features from the two spaces. The performances of the DGCNN model with these 4 cases are shown in Table 4 by retraining on ModelNet40 dataset.

**Table 4:** Performance comparison under different parameter combinations.

Condition	Accuracy (%)
$w_{xyz} = 1,w_{f} = 0$	91.7
$w_{xyz} = 0,w_{f} = 1$	92.4
$w_{xyz} = 0.5,w_{f} = 0.5$	93.1
ATSearch	93.3

As can be seen in the Table 4, compared to the methods of using a neighborhood on a particular space, simply changing $w_{xyz} ,w_{f}$ to 0.5 can improve the performance of the model because the neighborhood can obtain points from both 3D and feature space.

## Conclusion

In this study, ATSearch, a plug-and-play search method for point cloud analysis, is proposed. By adaptively combining 3D space searching with feature space searching, ATSearch can flexibly search point cloud neighborhoods, which enhances its ability to obtain information across regions. Moreover, the point attention block has a very low computational cost, so the whole structure is efficient. In the xperiment, our method changes only the model search method to ATSearch and then improves the performance of multiple models. In addition, the proposed method also shows good robustness to the chosen value of k. Neighborhood search technology is widely used in image processing and point cloud analysis, so ATSearch can be further applied to these fields, such as gesture recognition, emotion classification In the future, it will be worthwhile to consider how to accelerate the inference speed of our proposed network and the form of the weight generation function.

## Data Availability

All data included in this study are available upon request by contact with the corresponding author.
